# The role of angiogenic growth factors in the immune microenvironment of glioma

**DOI:** 10.3389/fonc.2023.1254694

**Published:** 2023-09-13

**Authors:** Zhengmao Ge, Qi Zhang, Wei Lin, Xiaofan Jiang, Yanyu Zhang

**Affiliations:** Department of Neurosurgery, Xijing Hospital, Fourth Military Medical University, Xi’an, China

**Keywords:** angiogenic growth factor, immune cell, glioma, immune modulation, tumor microenvironment

## Abstract

Angiogenic growth factors (AGFs) are a class of secreted cytokines related to angiogenesis that mainly include vascular endothelial growth factors (VEGFs), stromal-derived factor-1 (SDF-1), platelet-derived growth factors (PDGFs), fibroblast growth factors (FGFs), transforming growth factor-beta (TGF-β) and angiopoietins (ANGs). Accumulating evidence indicates that the role of AGFs is not only limited to tumor angiogenesis but also participating in tumor progression by other mechanisms that go beyond their angiogenic role. AGFs were shown to be upregulated in the glioma microenvironment characterized by extensive angiogenesis and high immunosuppression. AGFs produced by tumor and stromal cells can exert an immunomodulatory role in the glioma microenvironment by interacting with immune cells. This review aims to sum up the interactions among AGFs, immune cells and cancer cells with a particular emphasis on glioma and tries to provide new perspectives for understanding the glioma immune microenvironment and in-depth explorations for anti-glioma therapy.

## 1 Introduction

Angiogenic growth factors (AGFs) are a series of secreted cytokines that plays crucial roles in angiogenesis by interacting with their corresponding receptors. AGFs mainly include vascular endothelial growth factors (VEGFs), stromal-derived factor-1 (SDF-1), platelet-derived growth factors (PDGFs), fibroblast growth factors (FGFs), transforming growth factor-beta (TGF-beta) and angiopoietins (ANGs). Angiogenesis is one of the important hallmarks of tumors, in which process AGFs play essential roles (1). However, accumulating evidence indicates that the function of AGFs is not only limited to tumor angiogenesis but also involved in tumor progression via multiple mechanisms that go beyond their angiogenic role (2–6). In the tumor microenvironment (TME), immune cells reprogram and express immunosuppressive phenotypes that leads to escaping of tumor cells from host immune surveillance and attack, in which process AGFs can be involved (7, 8). Anti-angiogenic therapy by targeting AGFs to normalize tumor vessels has also been found to improve anti-tumor immunity. Combination of anti-angiogenic drugs and immunotherapy has become a canonical treatment for hepatocellular carcinoma, non-small cell lung cancer and renal cell carcinoma, but still, it has not always been successful (8–10). A better understanding of the tangled interplay among AGFs, immune cells and cancer cells in the TME is urgently needed to shed lights on finding new anti-tumor therapeutic avenues.

Glioma accounts for about 80% of all intracranial malignance (11). The 5-year survival rate for patients with glioblastoma (GBM) which is the most aggressive form of glioma is only 6.9% (12). Angiogenesis in glioma and GBM is particularly obvious among all tumors (13, 14). Anti-angiogenic therapy by targeting AGFs or their receptors has become a hot topic in the treatment of glioma during the past decades (15, 16). This therapy differs from the traditional anti-tumor therapies. It aims to inhibit tumor growth rather than directly attack tumor cells. Unfortunately, most clinical trials of anti-angiogenic treatment to glioma ended with patients showing serious side effects or no significant benefits (16, 17). Therefore, the anti-angiogenic treatment of glioma should be considered from a more comprehensive perspective than just focusing on the vessels. The microenvironment of glioma contains tumor cells, glial cells, immune cells, neurons, vasculature and extracellular matrix. The glioma microenvironment generates a pro-tumor dynamic with significant angiogenesis and immunosuppression (18). The innate immune system including natural killer (NK) cells, macrophages, microglia and neutrophils and the adaptive immune system together establish a well-regulated immunity for proper brain function (19). Besides angiogenesis, glioma exhibits strong immunosuppressive properties of the microenvironment (20, 21). The expression of most AGFs, represented by VEGFs, were significantly upregulated in glioma (22, 23).

**Figure 1:**
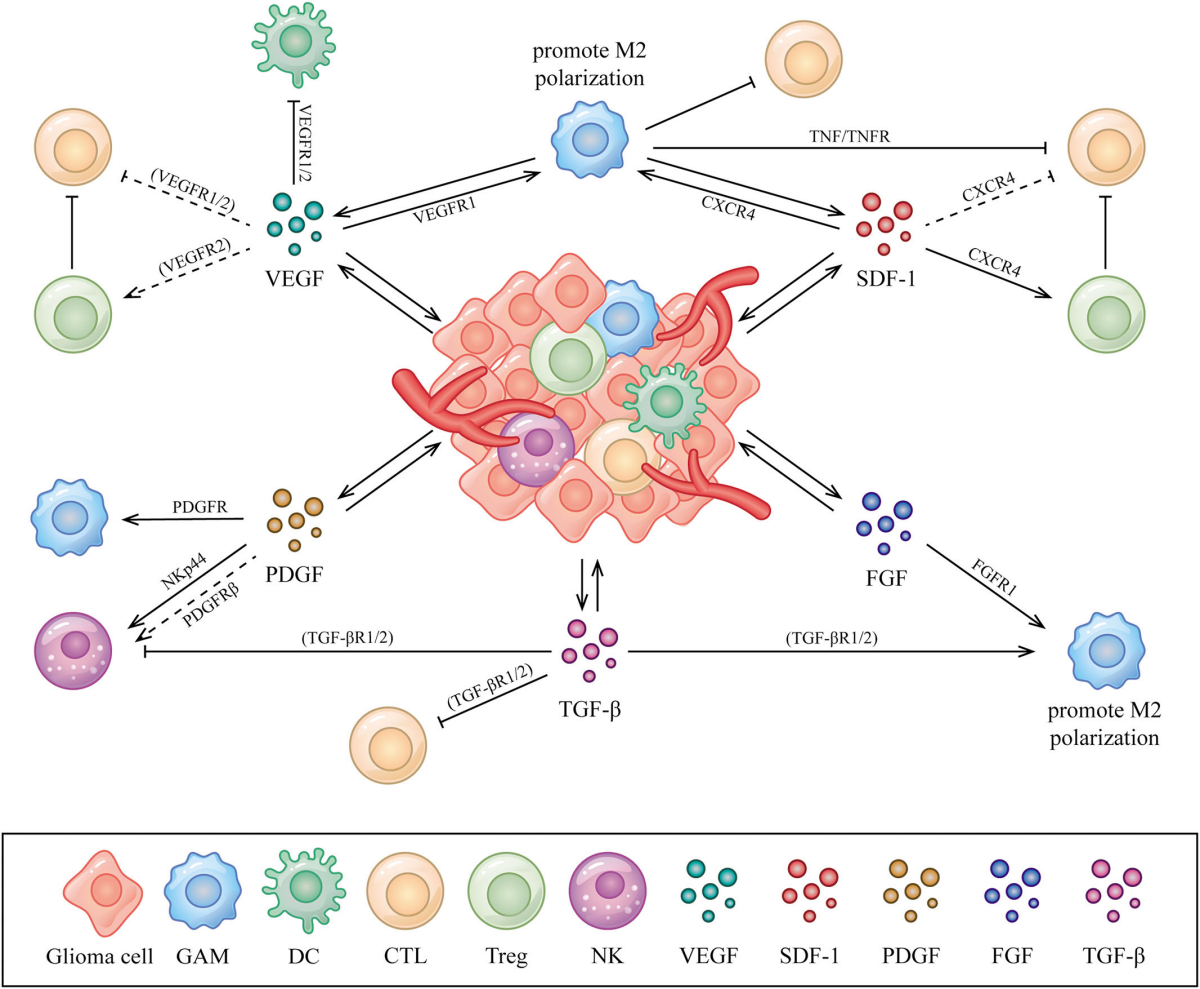
Interactions between AGFs, immune cells and tumor cells in the glioma microenvironment. AGFs including VEGFs, PDGFs, SDF-1, FGFs and TGF-β secreted by glioma or stromal cells could be upregulated in the glioma microenvironment, which acts on various immune cells including GAMs, DCs, T cells and NK cells to perform immunomodulatory functions mainly as immunosuppression. The directional arrow indicates effect of promoting. The suppressive arrow indicates effect of inhibiting. The dashed line indicates speculated associations that have not been confirmed in the glioma microenvironment but in other conditions.

Aberrantly expressed AGFs interacts with immune cells by binding to the related receptors that expressed could be one of the explanations of immunosuppression exhibited in the glioma microenvironment (24, 25). A comprehensive summary of the interactions between AGFs, immune cells and tumor cells in the glioma microenvironment is necessary, which may help us to better understand the connections and provide new perspectives for deeper explorations on anti-glioma treatment. In this article, we review the relations and interplay among AGFs, immune cells and tumor cells with a special focus on the glioma microenvironment (**Figure 1**).

## 2 VEGF

Vascular endothelial growth factors (VEGFs), alternatively referred to as vascular permeability factors, are a protein family related to angiogenesis. VEGFA, VEGFB, VEGFC, VEGFD, VEGFE (viral VEGF), VEGFF (snake venom VEGF), placental growth factor (PlGF) and endocrine gland-derived vascular endothelial growth factor (EG-VEGF) together construct VEGF family (26). This family of growth factors correspond to a variety of receptors including the tyrosine kinase receptors, which are named as VEGFR1-3 respectively, together with neuropilin and co-receptors of heparan sulfate proteoglycan families (27). VEGFA/VEGFR2 is the most potent combination in angiogenesis. VEGFC and VEGFD bind primarily to VEGFR3 to induce lymph angiogenesis and developmental angiogenesis. In contrast, VEGFR1 primarily acts to attenuate angiogenic signaling (28). However, VEGFR1 and VEGFR3 can play an alternative role in promoting angiogenesis when VEGFR2 pathway is reduced (29). In physiological conditions, VEGF is expressed in early embryos, various vascular tissues, heart, kidney, skeletal muscle, endocrine glands and other tissues, which play major roles in angiogenesis, vessel permeability and extracellular matrix degeneration.

In the course of tumorigenesis, VEGF is primarily derived from tumor cells and can also be secreted by smooth muscle cells, keratinocytes, neutrophils, platelets, macrophages, endothelial cells and fibroblasts (30, 31). VEGF expression can be elevated by activation of oncogenes such as Ras, stimulation of certain cytokines and growth factors including PDGF, epidermal growth factor (EGF), tumor necrosis factor-α, interleukin-1, interleukin-6 and HIF-1 (32). One of the typical pathological features of GBM is abundant angiogenesis which is associated with VEGF closely. A recent study reported that the expression level of VEGF is an independent risk factor for glioma prognosis (33). Studies have found that GBM cells express and secrete VEGFA protein, which was regulated by protein disulfide isomerase A4 in GBM cells (34, 35). Another study of GBM has shown that VEGFA was dramatically overexpressed while no significant change of VEGFB expression was detected in GBM patients (36). However, anti-VEGF therapy alone has not shown significant advantages in glioma treatment in patients. More and more emphasis on the function of VEGF of its immune effect has been shown. VEGFA was considered as a core negative gene affecting immune activity in the GBM microenvironment (37). VEGFRs are expressed both on the surface of immune cells and vascular endothelial cells. In GBM tissues, VEGFR1 is mainly expressed on tumor cells and tumor-associated macrophages. VEGFR2 is expressed mostly on tumor stem cells, regulatory T cells (Tregs) and vessel endothelial cells (38, 39). Due to the ability to interact with VEGF receptors expressed on immune cells, VEGF is able to exert great influences in the glioma immune microenvironment (40).

The primarily discovered immunosuppressive function of VEGF is to inhibit dendritic cell (DC) maturation, which causes less tumor antigen presentation and results in a potential immune avoidance of the tumor (41). VEGFR1 and VEGFR2 are expressed on DCs. VEGFR1 can regulate maturation of DC while VEGFR2 can control the function of mature DC (42, 43). It has been confirmed that VEGFR1 is a main receptor for VEGF-related suppression of DC maturation. When VEGF and PlGF binds to VEGFR1, the downstream signal transmission leads to blockade of the nuclear factor kappa-B (NF-κB) activation in hematopoietic progenitor cells of bone marrow which in turn affects the early stage of bone marrow/DC differentiation (42, 43). Researchers injected GL261 cells overexpressing Vector or VEGFC into the striatum of WT mice and confirmed that tumor-associated meningeal lymphangiogenesis are enhanced by VEGFC, which facilitates transportation of DCs from brain tumors to deep cervical lymph nodes (dCLNs) through CCL21/CCR7 signaling pathway. VEGFC overexpression in tumors induces a stronger immune response followed by the application of anti-PD-1 or anti-CTLA-4 treatment, in which the tumors displayed decreased tumor volumes and tumor weight and showed longer survival compared to the Vector group in the mice (44). In addition, it has been demonstrated that the association of anti-angiogenic therapeutic regimen with DC vaccination suppressed glioma progression in rats via stimulating immune response, suppressing glioma stem-cell-like cell development and inhibiting angiogenesis-related protein expressions such as ICAM-1, VCAM and VEGFs (45).

Tumor-associated macrophages (TAMs) perform a considerable function in the immune microenvironment of cancer. In glioma, they are named as glioma-associated microglia and/or macrophages (GAMs). GAMs usually contain macrophages recruited from circulating monocytes and microglia which arises from myeloid progenitors and resides in the brain for a long time (46). GAMs exhibited significant diversity as well as plasticity and showed distinctive phenotypes, which are ascribed to inflammatory (M1) or alternative (M2) polarized secretory forms (47). M1-type macrophages can increase the number of activated NK cells, while M2-type macrophages not only can inhibit CD8+ T lymphocyte proliferation but also upregulate the inhibitory receptor expression of CTLA-4 and PD-1 (48). In a similar way, M2-type GAMs, the major type of GAM, resulted in tumor progression by producing anti-inflammatory cytokines and growth factors, which inhibit the host immune reaction (49). VEGFA/VEGFR1 is one of the most important ligand-receptor pairs associated with recruitment of monocytes/macrophages to form GAMs in a specific TME regions around glioma. Macrophages in gliomas were differentiated from VEGF producing monocytes. The number of macrophages was positively correlated with VEGF expression (50). Meanwhile, macrophages upregulate VEGFR1 expression and promote formation of GAMs (51). Under ischemia, hypoxia or in the absence of nutrients, VEGF expression are stimulated in macrophages in tumors (52). Through activation of PI3K/Akt/Nrf2 pathway in GBM cells, VEGF produced by M2 GAMs promotes GBM cell stemness, proliferation, epithelial-mesenchymal transition (EMT), and temozolomide resistance (51, 53). VEGF downregulation caused by mitochondrial damage in tumor cells resulted in an increased rate of M1/M2 macrophages both *in vivo* and *in vitro*, which enhances the TAM effects to strengthen the immunity to against tumors in the microenvironment (54).

Tregs which express VEGFR2 on their surfaces are highly suppressive, which play a vital role in immune tolerance to tumors (38). Both Treg recruitment and cytotoxic T lymphocyte (CTL) depletion can happen at the same time under the joint action of VEGF and VEGFR1/2 (55, 56). A study revealed that VEGFA blockade can enhance T-cell transfer therapy by increasing the amount of pmel-1 T cell infiltration in a melanoma mouse model (57). Bevacizumab, an anti-VEGF humanized murine monoclonal antibody, is effective in inhibiting GAMs and Tregs and increasing CTL infiltration, thereby restores the supportive immune microenvironment in glioblastoma (58). The relationship between VEGF and T cells in glioma is also reflected in the blood vessel normalization of VEGF blockade, which enhances the effectiveness of CAR-T therapy in glioblastoma models in mice (59). Moreover, VEGFRs peptide vaccination can activate CTLs which ultimately kill tumor cells, endothelial cells and Tregs expressing VEGFR1/2 in primary glioblastomas patients in clinical trial (60).

In summary, VEGFs in glioma are mostly acting as immunosuppressive factors in the microenvironment, which help tumor cells to avoid immune surveillance and immune cell killing. VEGFs possess regulatory effects in the glioma immune microenvironment involving inhibition of DC maturation, amassment of GAMs and controlling infiltration of T lymphocytes. It is worth noting that not all types of VEGFs cause immunosuppression in the glioma microenvironment. VEGFC, for example, can enhance radiotherapy efficacy and anti-tumor immunity in gliomas when combined with VEGFR2. This effect was ascribed to the CCL21-dependent CD8+ T cell activation and DC trafficking (61). Tumor-associated dendritic cells (TA-DCs) suppress anti-tumor immune responses, but when treated with inflammatory molecules, TA-DCs gain the ability to reactivate T cells (62).

## 3 SDF-1

Stromal-derived factor-1 (SDF-1), also referred to C-X-C motif ligand 12 (CXCL12), is a primitive and conserved chemokine (63). SDF-1 is an acidic protein that can be expressed under both physiological and pathological conditions by diverse cells such as bone marrow cells, epithelial cells, endothelial cells and tumor cells. It is currently believed that there are three SDF-1 isoforms named as SDF-1α/β/γ respectively, among which SDF-1α showed the strongest effects on vessel sprouting and permeability (64). The major receptor of SDF-1 is C-X-C Motif Chemokine Receptor 4 (CXCR4), belonging to the family of G protein-coupled receptor (GPCR) which traverses the plasma membrane seven times. CXCR4 is widely distributed in smooth muscle cell precursors, endothelium precursor cells, endothelial cells, immature and mature hematopoietic cells, and also astrocytes, microglia and neurons in the central nervous system (CNS) of adults (65, 66). SDF-1 directly participates in angiogenesis by recruiting endothelial progenitor cells through coupling to CXCR4 on endothelial cells (67). SDF-1 may also promote angiogenesis indirectly, by stimulating endothelial cells to secrete proangiogenic cytokines such as CXCL1, CXCL8 and VEGF (68–70).

In glioma, studies of SDF-1 functions in promoting angiogenesis are relatively comprehensive, whereas here we mainly discuss the role of SDF-1 in the glioma immune microenvironment and its direct effects on glioma progression. SDF-1 and CXCR4 were the most frequently expressed mRNA identified in 31 human astrocytic neoplasms (71). Enhanced SDF-1 and CXCR4 expression was observed not only in low-grade glioma such as oligoastrocytomas and astrogliomas, but also in areas of core necrosis and marginal infiltration of glioblastoma (71–75). CXCR4 is widely expressed on the surface of a variety of leukocytes, such as monocytes, macrophages, T cells, B cells, eosinophils and neutrophils. Thus, SDF-1 can participate in immune regulation such as leukocyte migration, leukocyte homing and lymphocyte recycling (65).

In the glioma microenvironment, SDF-1 is mainly derived from glioma cells and microglia and binds to CXCR4+ cells including microglia, macrophages, monocytes, endothelial cells and glioma cells themselves to initiate G-protein subunit dissociation, and the subsequent stimulation of mitogen-activated protein kinase (MAPK), phosphoinositide 3-kinase (PI3K) and phospholipase C (PLC) (63, 76–80). Independent of G proteins, it has been demonstrated that Janus kinase (JAK)2 and JAK3 pathways are activated by SDF-1-CXCR4, which allows the signal transducer and activator of transcription (STAT) molecule recruitment and activation to initiate transcription of multiple cancer-associated genes (80–83). A large number of aberrantly activated STATs, especially STAT3 and STAT5, were found to be actively involved in tumorigenesis, immune surveillance escaping and self-immunotolerance of tumor cells in glioma (83, 84).

Glioma cell released SDF-1 causes GAMs that expresses CXCR4 to polarize towards a M2-like phenotype which is anti-inflammatory and pro-tumorigenic (85). Specifically, a study on zebrafish showed that SDF-1β/CXCR-4b signaling is required for macrophage infiltration and microglia differentiation in the brain (86). As an important part of the glioma immune microenvironment, M2 type of GAMs are functioning in inhibiting T cells, promoting glioma cell EMT and invasion (85, 87, 88). When SDF-1 binds to macrophages or CD8+ T cells that express CXCR4, tumor necrosis factors (TNFs) and tumor necrosis factor receptors (TNF-Rs) will be secreted respectively to exert immunosuppressive function by delivering pro-apoptotic signals to T cells (89). In a syngeneic murine glioma model, both the number of Tregs and the expression of CXCR4 showed time-dependent upregulation, which may be one of the reasons for immune escape of glioma cells (90). The above processes result in local immunosuppression in the glioma microenvironment which leads to glioma progression ultimately. In glioma mice, combined therapy of anti-CXCR4 and anti-PD-1 reduced immunosuppressive leukocytes counts, advanced the CD4+/CD8+ T cell ratio and raised the amount of pro-inflammatory cytokines (91).

SDF-1 can directly promote glioma aggression. It is identified that SDF-1α activates focal adhesion kinase (FAK) and proline-rich tyrosine kinase 2 (Pyk2) signaling pathways to promote invadopodia formation which enhances glioma cell invasiveness by a study including 20 human glioblastoma specimens (76). In the optic pathway glioma, SDF-1 is expressed by numerous brain-derived cells including endothelial cells, entrapped axons and infiltrating microglia, which enhances optic glioma cell survival via CXCR4 receptor presented on glioma cells, whereas blocking CXCR4 inhibited glioma development *in vivo (* 92, 93). Notably, the aggressiveness of the hepatocellular carcinoma is enhanced under the effect of ionizing radiation through increasing SDF-1 expression by epigenetic regulation (94). X-ray irradiation as a component of the normative therapeutic regimens for GBM (95) was frequently shown to enhance SDF-1 expression. Enlightening by this phenomenon, it would be of significance to clarify whether radiotherapy (RT) induces epigenetic regulation of the SDF-1 promoter to increase the expression, thereby increases glioblastoma invasiveness. Thus, attentions regarding to the potential disadvantages of RT need to be brought to clinicians and researchers, and individualized treatment by SDF-1 inhibition could be a potential solution.

CXCR4 and CXCR7 as receptors for SDF-1 are broadly involved in tumorigenesis (96). Dysregulated SDF-1-CXCR4/CXCR7 signaling has been observed in a diversity of tumor types involving gliomas (97). SDF-1-CXCR4/CXCR7 signaling shows both pro- and anti-inflammatory effects in tumors. On one hand, SDF-1 can mediate plasmacytoid DC trafficking to the tumor region and Tregs homing to the bone marrow microenvironment (98). On the other, it can promote the entry of immune cells with inhibitory functions like immature DC into the TME while rejecting immune effector cells (99). Current studies showed that Tregs with CXCR4 overexpression can be seen in several types of cancers which could explain why Tregs are recruited by SDF-1 at the site of tumorigenesis. However, this has not been confirmed in glioma. Upregulation of CXCR4 expression has also been seen in glioblastoma-associated T cells, but whether glioblastoma employ a similar mechanism to affect T cell infiltration remains unknown (100).

## 4 PDGF

The platelet-derived growth factor (PDGF) family contains four PDGFs (PDGFA, PDGFB, PDGFC and PDGFD) which forms five different disulphide-linked dimers (PDGF-AA, PDGF-BB, PDGF-CC, PDGF-DD and PDGF-AB) and two tyrosine kinases receptors (PDGF receptor α and β) (101). PDGF triggers the receptor kinase activity by driving the dimer formation from monomeric PDGFRs. PDGFRαα can be stimulated by all types of active ligand molecules except for PDGF-DD. PDGFRαβ can be stimulated by PDGF-AB, PDGF-BB and PDGF-CC whilst PDGFRββ can be stimulated by PDGF-BB or PDGF-DD (102). The activated PDGFRs initiates PI3K/AKT/mTOR, RAS/MAPK/ERK and JAK/STAT3 signaling pathways, which guide cells to survive, proliferate or migrate. Signaling is eventually terminated by internalization and degradation of the active PDGFR dimers (103). PDGFs/PDGFRs are expressed in various cell types under physiological conditions. In normal conditions, PDGFs are produced by megakaryocytes and preserved in platelets, and are released from disintegrated platelets when blood clots. PDGFs can also be secreted by diverse cells such as osteoblasts, fibroblasts, vascular smooth muscle cells, endothelial cells, glial cells and neurons (104). Different PDGF isoforms exert diverse functions. These functions include but are not limited to promoting organ development, wound healing and inducing macrophage recruitment (105). In tumors, PDGFs were shown to modulate tumor microenvironment, tumor growth and metastasis by targeting stromal cells, vascular endothelial cells and malignant cells (106). Human glioma expresses all PDGFs and PDGFRs. PDGFRα is found to be expressed mostly in glioma cells, whereas PDGFRβ is expressed mainly by the stromal cells (107).

PDGFs/PDGFRs are closely relevant to the physiopathological processes of glioma cells and tumor microenvironment. The interaction between PDGFs and GAMs promotes glioma cell migration as well as creating an immunosuppressive milieu of the TME. PDGFs were shown to increase monocyte and macrophage infiltration and promote inflammation in gliomas. In a mouse glioma model, high-grade glioma was developed in connection with intratumoral macrophages infiltration, in which mice PDGFB was expressed under the control of glioneuronal-specific nestin promoter (108). In another study, by immunohistochemical stainings on human pediatric high-grade gliomas (HGG) tissues, PDGFB/PDGFRβ was found to be strongly associated with IBA 1+, which suggested a possible role of the PDGF signaling in the infiltration of TAM (IBA 1+) (109). PDGFD was shown to promote neuroinflammation and enhances macrophage infiltration by an *in vivo* study of the mouse after intracerebral hemorrhage (110). Matrix metalloproteinases (MMPs) are important enzymes involved in monocytes/macrophages infiltration. PDGFC and PDGFD were showed to stimulate monocyte migration by expressing MMPs (MMP-2 and MMP-9) in an *in vitro* study (111). PDGFs released by microglia can also affect glioma cell migration. A study on glioma mouse model showed that M2-polarized microglia rather than M2-polarized bone marrow-derived macrophage is the reason for the elevated expression of PDGFRβ on glioma cells and their increased ability of migration (107). Furthermore, another study showed that PDGFB as well as SDF-1α released by microglia are key factors that may induce the formation of invadopodia and stimulate cell migration by activating Pyk2 and FAK kinases in human glioblastoma (76).

NK cells are known to kill tumor cells through several pathways, among which the most important way is to identify surface marker molecules on target cells and induce apoptosis through secretion of cytotoxins and cytokines. Recently an important study showed that PDGFD can inhibit tumor growth by inducing immunoreceptor tyrosine-based activation motif (ITAM) signaling via NKp44, a NK cell receptor, which leads to the generation of anti-tumoral factors from NK cells (112). Further study reported that PDGFD contributes to IL-15-mediated NK cell survival other than its effector function via PDGFRβindependently from NKp44 (113). A recent study has confirmed that the transcriptional profile of NK cells stimulated by PDGFD can predict a better prognosis of patients with low-grade glioma, suggesting that NK cells may be more conducive to against tumors under the action of PDGF (114). These studies indicate that PDGFD not only promotes tumor growth and stromal response but also activates innate immune systems in response to tumors. Therapeutics that selectively targeting PDGFD pathway in NK cells may provide a new aspect in the immunotherapies (113).

In addition, PDGF-AB is found to inhibit dendritic cell maturation by upregulating C-type lectin-like receptor 2 (CLEC2), which can further induce the formation of Tregs. PDGFB is shown to be associated with CD4+ T cell inhibition by inducing jumonji domain-containing protein 6 in patients with chronic hepatitis B infection (115).

At present, PDGF/PDGFR inhibitors have not achieved satisfactory results in the treatment of glioma (116, 117). Although forementioned evidence suggested that PDGFs play important roles in inflammatory cell activation and migration and some signaling pathways in certain cells have been explored, the specific mechanisms in the context of glioma remains largely unclear. Studies on how PDGFs/PDGFRs interacts with NK cells and regulatory T cells in glioma microenvironment may be worth to know.

## 5 TGF-β

Transforming growth factor-beta (TGF-β) indicates a large superfamily which contains three TGF-β isoforms (TGF-β1, -β2 and -β3), as well as Activins, Nodal, bone morphogenetic proteins (BMPs), the growth differentiation factors (GDFs) and the müllerian inhibiting substance (MIS) (118). As a multifunctional polypeptide cytokine, TGF-β plays important roles in angiogenesis, cell proliferation, wound healing and immune regulation by binding to a hetero-tetramer of TGF-β1 and TGF-β2 receptor serine/threonine kinases (119, 120). TGF-β is classified as AGFs because of its pro-angiogenic effect through stimulating endothelial cell and cancer-associated fibroblast proliferation, migration and sprouting both directly or indirectly (121, 122).

Hereby we mainly review the function of TGF-β in immunity specially in the glioma microenvironment. TGF-β effects as tumor inhibitors at the beginning but promotes tumor progression in the late stages of tumorigenesis (123). The increased TGF-β expression is associated with higher malignant degree and poorer prognosis of glioma patients (124, 125). TGF-β is able to affect the activity of immunocytes to regulate immune responses in the TME (126). In malignant gliomas, especially in GBM, TGF-β can be released by tumor cells, Tregs, M2-type GAM and myeloid-derived suppressor cells (MDSC) (127–129). TGF-β has been reported to inhibit the anti-tumor immunoreaction and modulate the properties of both GBM cells and the stromal cells in TME (129, 130). In glioma patients with different WHO grades, TGF-β1 and TGF-β2 were found to be the major TGF-β isoforms secreted by glioma cells which are able to downregulate cellular adhesion molecule (CAM) expression on GBM endothelial cells to prevent T cell infiltration (131). TGF-β2 has been observed to mitigate the recognition of glioma cells by CD4+ T lymphocytes through reducing the expression of HLA-DR antigen on human glioma cells (132). One study on GBM patients reported that TGF-β helps GBM cells to escape from the recognition of immune effector cells by downregulating the NKG2D which is one of the receptors expressed on NK cells and CD8+ T cells (133). In another study, TGF-β1 can inhibit the NK cell recognition and killing of glioblastoma stem cells through TGF-βR1/2 and the Smad2/3 phosphorylation in NK cells (127). In addition, in the presence of TGF-β1 *in vitro*, GAMs can be recruited and polarized to the M2-phonotype by Smad and PI3K/AKT signaling pathway (134, 135).

As a multifunctional cytokine, TGF-β supports tumor progression in general and contributes to generating an immunosuppressive microenvironment in glioma. Overexpression of TGF-β and its effects on anti-tumor immune responses by interacting with T cells and NK cells have been recognized in GBM (130). Notably, higher cytoplasmic TGF-β1 levels were found to be related to longer survival of patients with astrocytoma (136). This phenomenon may need to be considered when employing TGF-β related therapy on glioma patients.

## 6 FGF

Fibroblast growth factors (FGFs) were originally discovered and named for its function of promoting fibroblast growth. It is a family consists of twenty-two members (FGFs1–23), of which the mouse FGF15 is an ortholog of human FGF19 and ten of these factors were identified in the brain (137, 138). Similar to other growth factors, FGFs exert their effects through activation of four specific receptors named fibroblast growth factor receptor (FGFR) 1-4, which are receptor tyrosine kinases (RTKs) (139). FGFs are essential for embryonic development, cellular proliferation, angiogenesis, and tissue repair (140, 141). Thus, FGF family is also considered as AGFs. Overexpression of FGFs/FGFRs or continuous activation of FGFRs caused by chromosomal translocations has been found in a variety of tumors leading to up-regulation of downstream signaling involving NF-κB, STAT, RAS-MAPK, PI3K-AKT and phospholipase Cγ (PLCγ) pathways (142). Sustained activation of the above signaling pathways eventually leads to uncontrollable cell division, angiogenesis and EMT (143).

FGFs are differentially expressed in different types of tumors and play important roles through paracrine or endocrine signaling. In glioma tissues, higher levels of FGF1 and FGF2 were detected compared to normal brain tissues (144). It was reported that mutations of the FGFR1 kinase domain were found in human GBM tissue and the expression of FGFR1, FGFR3 and FGFR4 was increased in glioma (145–148). In contrast, FGFR2 expression is low or undetectable in high-grade astrocytomas whereas it is abundantly expressed in normal brain tissues (149). A recent study revealed that FGFR1 on macrophages are activated by glioma cell-derived FGF20, which inhibits β-catenin degradation through phosphorylating glycogen synthase kinase 3β (GSK3β) and subsequently suppresses macrophage polarization to the M1 phonotypes *in vitro* (150). In addition, studies show that FGF2 originated from TAMs in MC38 tumors plays a significant role for the transition of TAMs to a pro-tumorigenic M2 phenotype (151). FGF signaling pathway including FGFR1, FGFR4 and FGFR23 was shown to be involved in recruiting immunosuppressive cells, regulating M2 polarization of GAMs and T cell exhaustion in gliomas (152). These findings may serve as a basis for targeting FGF signaling to regulate immune response of gliomas. Nevertheless, the effects of other FGFs on the immune microenvironment of gliomas are currently unclear.

## 7 Angiopoietins

Angiopoietins (ANGs) are a group of angiogenic growth factors belonging to the angiopoietin family, which consists of ANG1-4, the vascular endothelial-protein tyrosine phosphatase (VE-PTP) and the associated receptor tyrosine kinase Tie1 and Tie2 which are mainly expressed on the surface of endothelial cells and hematopoietic cells (153–155). ANG1 and ANG2 are the most studied factors functioning in regulating vascular integrity in an opposite way (156). The ratio of ANG2/1 was increased in mesenchymal cells from GBM specimens compared to normal human cerebrovascular pericytes (157). Upregulation of ANG2 expression was observed in GBM mouse model (158). The abnormal expression of ANG2 is considered to be the other major cause besides VEGF for the massive heterogeneous angiogenesis happened in gliomas (159). In addition, ANG2 has been shown to promote cell proliferation, invasiveness and malignant transformation of gliomas both *in vitro* and in mouse models (160, 161). In the immune aspects, ANG2 is considered as immunosuppressive in tumors by recruiting MDSC, regulatory T cells and monocytes (162).

Based on the study by analyzing the TCGA cohort, ANG2 was shown to be involved in immune regulation in TME by interacting with neutrophils, macrophages, NK cells and mast cells (163). In some kinds of solid tumors, angiopoietins were reported to be associated with immune cells including TAMs, NK cells and neutrophils, but whether it effects in a similar way in the glioma microenvironment requires further confirmation (164, 165). Available evidences showed that Tie2 was expressed in immunocytes including DCs, TAMs, T lymphocytes, neutrophils and mast cells, combining with the findings that ANG2 was expressed aberrantly in gliomas, provide us a feasible theoretical basis for further studying the ANG and immune cell interactions in the glioma microenvironment (62, 166–169).

## 8 Concluding remarks and perspectives

Angiogenesis is one of the hallmarks of cancer (1). Gliomas, particularly high-grade gliomas including GBM, exhibits enhanced angiogenesis and immunosuppression (170, 171). Standard treatment fails to improve glioma patient survival in an efficient way, when combining with anti-angiogenic therapy, the clinical results were still unsatisfactory (172, 173). After a period of usage, resistance was shown in anti-angiogenic therapy which develops compensatory pathways to maintain glioma angiogenesis and growth (16, 174).

In recent years, a number of anti-vascular treatment regimens targeting AGFs or AGF receptors have been proposed in glioma. The therapeutic agents targeting VRGF are the mostly studied. Bevacizumab (BEV), the only VEGF-targeting drug approved by the US Food and Drug Administration (FDA) for GBM patients, has not been shown to improve overall survival (OS) in Phase III clinical trial unlike the results from the colorectal cancer trials which acquired significant effects in OS improvement (16, 175). As well, BEV also showed no significant effects in another Phase II clinical trial for patients with WHO grade 2 and grade 3 gliomas (176). Incidentally, the AGF receptors such as VEGFR, PDGFR, FGFR and TIE are all belong to the receptor tyrosine kinases (RTKs). Imatinib, which is the first kinase inhibitor received FDA approval in 2001 has been widely used in the treatment of leukemia and gastrointestinal tumors (177–179). Whereas, the efficacy of imatinib in the clinical treatment of glioma needs further validation (180–182). In addition, AMD3000 as a CXCR4 (SDF-1 receptor) antagonist, has been shown to have therapeutic potential in glioma *in vivo* and *in vitro*. It can ultimately improve the glioma immune microenvironment by reducing CXCR4+ monocyte myeloid-derived suppressor cell infiltration and inducing immunogenic cell death (183). Such therapies are often proposed to target tumor angiogenesis initially. Worth to note that due to the existence of blood-brain barrier, appropriate drug carriers could be considered to achieve better therapeutic effects when applying anti-AGFs and related therapy (184). Significantly, anti-AGFs or their corresponding receptors with combined therapeutic methods, for example immunotherapy, may be helpful for glioma treatment.

AGFs were significantly upregulated in gliomas (22). In the glioma microenvironment, there is evidence showing upregulations of VEGFs, SDF-1, PDGFs, TGF-β, FGFs and ANGs that we have reviewed, which lead to pathological effects such as tumor growth and dysfunction of blood vessels. Besides angiogenesis, the function of AGFs in tumor aggression is also reflected in immune regulation (115). Studies have shown that many of these AGFs can be the important causes of immunosuppression in the glioma microenvironment. Combination of anti-angiogenic drugs and immunotherapy has become one of the standard therapeutic regimens for multiple cancers (8–10). At present, the role of AGFs and their corresponding receptors in glioma immune microenvironment have not been comprehensively studied. Elucidating the interplay between AGFs and immunocytes in the glioma microenvironment can be a crucial step for improving the anti-glioma therapy. Here we summarize the AGF receptors expressed on immune cells and patient glioma cells, which could be of certain significance for the research of AGFs in the immune microenvironment of gliomas (**Table 1**).

**Table 1:** Angiogenic growth factor receptors on immune cells and patient glioma cells.

Cell types	Receptors	References
dendritic cells	VEGFR1, VEGFR2, VEGFR3, CXCR4, TGF-βR2, TGF-βR1*, FGFR1*, FGFR4*	(42, 43, 185–187)
monocytes/macrophages	VEGFR1, VEGFR2, VEGFR3, PDGFRα, PDGFRβ, CXCR4, TGF-βR1, TGF-βR2, FGFR1, Tie2	(43, 110, 166, 186, 188–192)
microglias	VEGFR1, VEGFR2, VEGFR3**, PDGFRα, PDGFRβ, CXCR4, TGF-βR1**, FGFR1, FGFR2, FGFR3, Tie2	(193–201)
CD4+ T cells	VEGFR1, VEGFR2, CXCR4, TGF-βR2, FGFR1, VEGFR3 *, PDGFRα*, TGF-βR1*	(38, 202–205)
CD8+ T cells	VEGFR1, VEGFR2, CXCR4, TGF-βR2, VEGFR3*, PDGFRβ*, TGF-βR1*	(186, 204, 206, 207)
natural killer cells	VEGFR3, CXCR4, PDGFRβ**, TGF-βR2, TGF-βR1*, FGFR1*, Tie-1*	(113, 186, 208, 209),
neutrophils	VEGFR1, VEGFR2, CXCR4, Tie2, VEGFR3*, PDGFRα*, TGF-βR1*, TGF-βR2*, FGFR1*, FGFR2*, FGFR4*	(168, 210–212)
mast cells	VEGFR1, VEGFR2, Tie1, Tie2	(213)
patient glioma/GBM cells	VEGFR1, VEGFR2, VEGFR3, PDGFRα, PDGFRβ, CXCR4, CXCR7, TGF-βR1, TGF-βR2, FGFR1, FGFR3, FGFR4, Tie2	(71, 214–220)

* The expression is suggested by the HPA database from https://www.proteinatlas.org/. No relevant literature report was retrieved in PubMed.

** The receptor is expressed with conditions.

With the deepening in neuroimmunology research, the effect of AGFs on immune cells in the glioma microenvironment attracted attentions (221). It is worth noting that TAM content varies in different types of gliomas, in which higher levels of TAM was detected in high-grade gliomas (versus low-grade), recurrent GBM (versus primary), and IDH-WT GBM (versus IDH-mutant) (222). Therefore, it is most likely that there is a certain correlation between the prognosis of glioma with TAM whose recruitment and function can be influenced by AGFs to some extent. We noticed that the AGF receptors expressed on microglia, as a type of resident and unique macrophages comprising 10% of brain cells in the CNS (223), are mostly the same as those expressed on macrophages. Nevertheless, we observed that there are more types of FGFRs on microglia than macrophages which may deserve further explorations. Up to now, researchers have refined the different origins of macrophages in the CNS to be monocyte-derived macrophage-derived TAMs (TAM-MDM) and microglia derived TAMs (TAM-MG). It has been found that the two types of cells tend to show different characteristics in distribution and function, which would be meaningful for the GAM study (222). In addition, it has been confirmed that mature myeloid-derived mast cells can enter the CNS from the periphery, which can be used as a prognostic factor for glioma patients. The mechanisms of how mast cells affect glioma immune microenvironment is still unclear (224–226). Worth to note, besides common immune cells, endothelial cells have also been confirmed to directly participate in immunosuppression in the microenvironment of glioma (227, 228).

In exploration of anti-angiogenic treatment, the effects on immune regulation should be considered. Besides the AGFs discussed in this review, it is worth mentioning that angiogenesis-associated factors such as sonic hedgehog (Shh), matrix metalloproteinases (MMPs) and microRNAs (miRNAs) may also play a part in the glioma immune microenvironment. Studies on the regulation of AGFs in the glioma immune microenvironment will help further understanding the disease and may reveal potential clinical treatment for gliomas.

## Author contributions

ZG: Conceptualization, Writing – original draft, Writing – review & editing, Visualization. QZ: Visualization, Writing – original draft. WL: Supervision, Writing – review & editing. XJ: Supervision, Writing – review & editing, Conceptualization, Funding acquisition. YZ: Conceptualization, Funding acquisition, Supervision, Writing – review & editing, Project administration, Writing – original draft.
